# A Solar-Heated Phase Change Composite Fiber with a Core–Shell Structure for the Recovery of Highly Viscous Crude Oil

**DOI:** 10.3390/polym17020135

**Published:** 2025-01-08

**Authors:** Chenxin Lin, Yifan Wang, Cenyu Liu, Kaiyue Meng, Endong Chang, Xiaowen Wu, Jiancheng Wang

**Affiliations:** 1 Engineering Research Center of Ministry of Education for Geological Carbon Storage and Low Carbon Utilization of Resources, Beijing Key Laboratory of Materials Utilization of Nonmetallic Minerals and Solid Wastes, National Laboratory of Mineral Materials, School of Materials Science and Technology, China University of Geosciences, Beijing 100083, China; 2 Binzhou Institute of Technology, Weiqiao-UCAS Science and Technology Park, Binzhou 256606, China

**Keywords:** crude oil spill, phase change material, photothermal conversion, coaxial electrospinning, hydrophobic modification

## Abstract

Due to the high viscosity and low fluidity of viscous crude oil, how to effectively recover spilled crude oil is still a major global challenge. Although solar thermal absorbers have made significant progress in accelerating oil recovery, its practical application is largely restricted by the variability of solar radiation intensity, which is influenced by external environmental factors. To address this issue, this study created a new composite fiber that not only possesses solar energy conversion and storage capabilities but also facilitates crude oil removal. PF@PAN@PEG was obtained by coaxial electrospinning processing, with PEG within PAN fibers, and a coating layer was applied to the fiber surface to impart oleophilicity and hydrophobicity. PF@PAN@PEG exhibited a high latent heat value (77.12 J/g), high porosity, and excellent photothermal conversion and oil storage capabilities, significantly reducing the viscosity of crude oil. PF@PAN@PEG can adsorb approximately 11.65 g/g of crude oil under sunlight irradiation. Notably, due to the encapsulation of PEG, PF@PAN@PEG can continuously maintain the crude oil at a phase change temperature by releasing latent heat under specific conditions, effectively reducing its viscosity with no PEG leakage at all. When solar light intensity varied, the crude oil collection efficiency increased by 21.99% compared to when no phase change material was added. This research offers a potential approach for the effective use of clean energy and the collection of viscous crude oil spill pollution.

## 1. Introduction

As the offshore crude oil exploitation and transportation industries continue to develop, the occurrence of offshore oil spill accidents has gradually increased [1,2]. Crude oil spills not only cause long-term and severe damage to marine ecosystems [3,4,5], but also result in a significant impact on the coastal economy [6,7,8]. Therefore, developing technology for the adsorption of offshore crude oil has become particularly crucial.

Various advanced porous absorbents with hydrophobic and lipophilic channels are considered as key technologies for offshore crude oil adsorption due to their recyclability and strong oil adsorption capacity. These adsorbents include natural materials (e.g., cellulose and cotton) [9,10,11,12,13,14] and polymers (e.g., commercial sponges and various types of gels) [15,16,17,18,19,20]. Among these, the porous structure and large specific surface area of nanofibers give them rapid response and high adsorption capacity characteristics, making them an ideal material in the field of offshore crude oil adsorption.

However, these adsorbents are only applicable to the recovery of low-viscosity oil and cannot effectively adsorb viscous crude oil. The high viscosity of viscous crude oil significantly hinders the diffusion of viscous crude oil into the pores of adsorbents. However, the viscosity of viscous crude oil has obvious temperature sensitivities, and its viscosity decreases sharply at higher temperatures, thereby improving the fluidity of the crude oil [21,22]. Therefore, imparting heating and thermal storage capabilities to adsorptive materials can significantly enhance their performance in offshore viscous crude oil adsorption.

Compared with two artificial heating methods, Joule heating [21,23,24,25,26,27] and magnetic field heating [28,29,30], collecting solar energy and converting it into thermal energy is a low-cost and simple method [31,32,33,34,35]. However, solar energy is highly influenced by weather variations, and it is difficult to provide a continuous and stable heat source when the light intensity is weak. Phase change materials (PCMs), as functional materials, absorb or release large amounts of latent heat through changes in their physical state (e.g., solid–liquid, liquid–gas). During the phase change process, the temperature of the material remains basically constant. Their high energy density and excellent temperature stability make them outstanding for thermal storage applications. By storing latent heat through phase change materials, the reliance of adsorbent materials on sunlight can be effectively reduced [36,37,38].

In this work, encapsulated phase change fibers were prepared by coaxial electrospinning and were further modified by a hydrophobic treatment to produce a photothermal phase change, oil-adsorbing fiber material (PF@PAN@PEG). When light was strong, the viscosity of the viscous crude oil around PF@PAN@PEG decreased sharply due to the photothermal effect, thereby accelerating the adsorption of the viscous crude oil; in the meantime, the phase change material stored thermal energy. When the sunlight intensity weakened, the latent heat stored in the phase change material was released, maintaining the phase transition temperature. In addition, by integrating with a peristaltic pump and tubing, PF@PAN@PEG can efficiently and continuously collect viscous crude oil through capillary pressure under solar irradiation conditions.

## 2. Materials and Methods

### 2.1. Materials and Reagent

Polyacrylonitrile (PAN, Mw ≥ 250,000) was purchased from Tao Chemical Industry. N,N -Dimethylacetamide (DMAC, AR, 99%) was purchased from Aladdin Biochemical Technology Co., Ltd. (Shanghai, China). Hydroxylated multi-walled carbon nanotubes (MWCNTs, TF-006H) were purchased from Suzhou Carbon Graphene Technology Co., Ltd. (Suzhou, Jiangsu, China). Polyethylene glycol (PEG-10,000, Mw ≥ 10,000) was purchased from Shanghai Ron Reagent Co., Ltd. (Shanghai, China). The surfactant (Chemours FS-3100) was purchased from the Chemours Company (Shanghai, China). N-Hexane (GC, >98%) was purchased from Shanghai McLean Biochemical Technology Co., Ltd. (Shnaghai, China). 1H, 1H, 2H, 2H-Dperfluorodecyltrimethoxysilane (PFDTMS, 97%) was purchased from Shanghai Yien Chemical Technology Co., Ltd. (Shanghai, China). Polydimethylsiloxane and the curing agent (DC184/SYLGARD184) were purchased from Dow Corning Co., Ltd. (Shanghai, China). Fe_3_O_4_ nanoparticles (Fe_3_O_4_ nPs; the average particle size was 20 nm) were purchased from Shanghai Yaoyi Alloy Materials Co., Ltd. (Shanghai, China). Crude oil was provided by the Ocean University of China (Qingdao, Shandong, China).

### 2.2. Preparation of Composite Fibers

#### 2.2.1. Preparation of the PAN Fiber

PAN fibers doped with MWCNTs-OH were prepared by electrospinning. Firstly, PAN, MWCNTs-OH, and Chemours FS-3100 were dissolved in DMAC and stirred at 60 °C for 12 h, and then left to stand for 12 h to make a homogeneous spinning solution. The concentration of PAN was 0.14 g/mL, MWCNTs was 0.014 g/mL, and Chemours FS-3100 was 1 μL/mL. The spinning solution was injected into a 5 mL syringe in the self-constructed electrospinning device, and the distance between the needle and the drum collector was 15 cm. The feeding rate was 1.5 mL/h, and the applied voltage was 15 kV. The electrospinning environment was maintained at 33 °C with a relative humidity of 20%. After the electrospinning process was completed, the membrane was placed in an oven at 60 °C for 24 h to remove the residual solvent and obtain the PAN fiber membranes.

#### 2.2.2. Preparation of the PAN@PEG Fiber

PAN@PEG was prepared by coaxial electrospinning. The spinning solution prepared in Section 2.2.1 was used as the shell layer spinning solution. PEG-10,000 was dissolved in DMAC and stirred at 70 °C for 30 min, and then left to stand for 30 min to make a homogeneous spinning solution, which was used as the core layer spinning solution. The concentration of PEG-10,000 was 0.45 g/mL. The two spinning solutions were injected into two 5 mL syringes in the self-constructed electrospinning device, which were then fed into a coaxial needle through two independent injection pumps. The distance between the needle and the drum collector was 15 cm. The feeding rate of the shell layer was 1.2 mL/h, the feeding rate of the core layer was 0.5 mL/h, and the applied voltage was 15 kV. The electrospinning environment was maintained at 33 °C with a relative humidity of 20%. After the electrospinning process was completed, the membrane was placed in an oven at 60 °C for 24 h to remove the residual solvent and obtain the PAN@PEG fiber membranes.

#### 2.2.3. Modification of the PF@PAN@PEG Fiber

A hydrophobic modification solution was prepared with n-hexane as the solvent. The concentrations of PDMS, crosslinker, PFDTMS, and Fe_3_O_4_ nPs were 10 mg/mL, 1 mg/mL, 1 mg/mL, and 1 mg/mL, respectively. The prepared solution was sonicated for 30 min to form a homogeneous mixture. The PAN@PEG fiber membrane was immersed in the hydrophobic modification solution and followed by an ultrasonic treatment for 10 min. Then, the membranes were shaken on an oscillation shaker for 6 h, allowed to stand for 6 h, and subsequently cured at 60 °C for 12 h in an oven to obtain PF@PAN@PEG, in which the meaning of P in PF is the added polymer, and the meaning of F is the added Fe_3_O_4_ nPs.

### 2.3. Characterization

A scanning electron microscope (SEM, Zeiss Supra-55, Baden-Württemberg, Germany) was used to observe the morphology of the samples. The element distribution in the samples was determined via elemental dispersive spectroscopy (EDS) mapping and obtained on the same instrument. The core–shell structure of the samples was observed using a transmission electron microscope (TEM, JEOL JEM-F200, Musashino, Akishima, Tokyo, Japan). Fourier transform infrared spectroscopy (FT-IR, Nicolet iS20, Thermo Fisher Scientific, Waltham, MA, USA) was used for the characterization of the samples. The UV–vis–NIR absorption spectra (200–800 nm) of the samples were measured by an ultraviolet spectrophotometer (UV-2600, Shimadzu Instruments (Suzhou) Co., Ltd., Suzhou, Jiangsu, China). The properties of phase change materials were analyzed by differential scanning calorimetry (DSC, DSC 214 Polyma, NETZSCH, Bavaria, Germany) between 25 °C and 85 °C at a rate of 10 °C/min in N_2_ atmosphere. The water contact angle (WCA) was measured under ambient conditions using a contact angle analyzer (SDC-200S, Shanghai Yuchen Testing Instrument Co., Ltd., Shanghai, China). Solar illumination was simulated by a xenon lamp light source (HF-GHX-XE-300, Shanghai Hefan Instrument Co., Ltd., Shanghai, China) with a wavelength range consistent with the standard solar spectrum of AM 1.5 G. The light intensity was measured using a solar power meter (SM206-SOLAR, Shenzhen Xinbaorui Instrument Co., Ltd., Shanghai, China). Temperature changes in the samples under light exposure were recorded using a multi-channel data logger (TP720, Shenzhen Topray Electronics Co., Ltd., Shenzhen, China). A viscometer (DV2T, Thermo Fisher Scientific, Waltham, MA, USA) was used to measure the viscosity of the crude oil.

### 2.4. Photothermal Performance Experiment

The photothermal properties of the samples were analyzed by a xenon lamp and a multi-channel data logger. Solar heating experiments were conducted using the xenon lamp as a solar simulator (power density: 1.0 kW·m^2^, equivalent to the light intensity of 1 sun). The temperature–time curves of samples under illumination were recorded by the multi-channel data logger.

### 2.5. Crude Oil Adsorption and Recovery Experiment

#### 2.5.1. Test of Static Crude Oil Adsorption

A vessel of viscous crude oil was set on an electronic balance during the static adsorption test. The PF@PAN@PEG sample (20 mm × 25 mm × 1 mm) was placed with its bottom on the surface of the crude oil for 15 min, while simultaneously being irradiated with a xenon lamp to allow for complete adsorption. Afterward, the saturated sample was suspended for 10 min to allow any adhered viscous crude oil to completely drip off. Next, a glass plate was used, and a force of 15 N was applied to extrude the stored crude oil from the sample. The adsorption capacity of crude oil per unit mass of the sample was calculated according to Equation (1):

(1) $$Q=\frac{\left(m_{1}-m_{2}\right)}{m_{0}}$$

where *m*_0_ is the sample mass (g), *m*_1_ is the mass of the sample after the adsorption of viscous crude oil (g), and *m*_2_ is the mass of the sample after the viscous crude oil was released (g).

#### 2.5.2. Test of Continuous Crude Oil Recovery

The PF@PAN@PEG sample (20 mm × 25 mm × 1 mm) was floated on the crude oil (~20 mL) and deionized water (30 mL) in a custom-made device to simulate the process of continuous viscous crude oil adsorption at sea. The PF@PAN@PEG was connected to the collector via tubing with a peristaltic pump running at a speed of 150 r/min. The outlet of the tubing was connected to a petri dish to collect the crude oil. The solar heating simulation experiment was conducted using a xenon lamp (power density: 1.0 kW m^2^, equivalent to 1 sun’s light intensity).

## 3. Results and Discussion

### 3.1. Morphology and Structure of Samples

**Figure 1:**
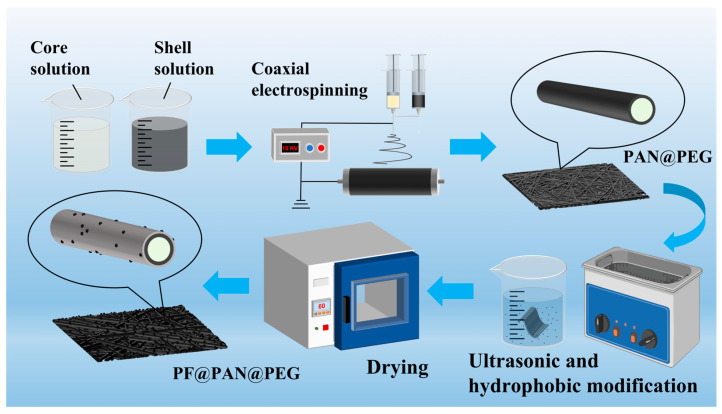
Schematic diagram of the preparation process of PF@PAN@PEG.

The preparation process of PF@PAN@PEG is shown in Figure 1. In summary, the novel PF@PAN@PEG composite PCM was prepared by pre-electrospinning PAN@PEG coaxial fibers using a coaxial electrospinning method, encapsulating PEG within PAN, and then imparting hydrophobic and oleophilic properties to it by immersing it in the hydrophobic modifier, further enhancing its light absorption capabilities.

**Figure 2:**
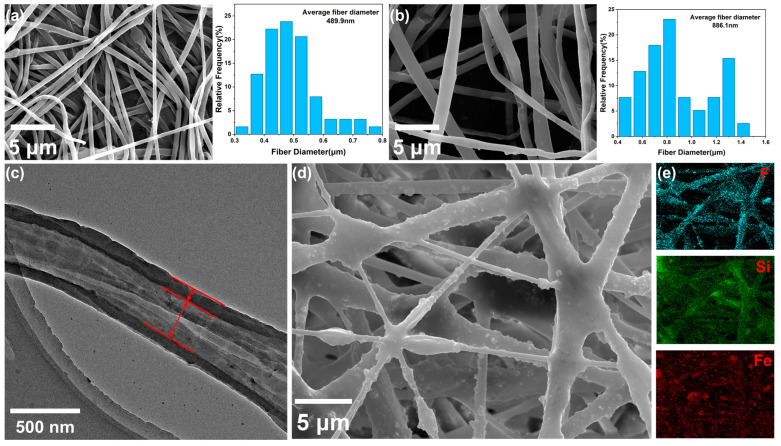
(**a**) SEM image and average fiber diameter of PAN; (**b**) SEM image and average fiber diameter of PAN@PEG; (**c**) TEM image of PAN @ PEG, where red arrows represent the core and shell layers of the fiber; (**d**) SEM image of PF@PAN@PEG; (**e**) corresponding EDS image of PF@PAN@PEG.

As shown in Figure 2a,b, the SEM images of PAN and PAN@PEG reveal that both fibers have smooth surface structures. It can be seen that the average diameter of the PAN fibers was 489.9 nm, while the diameter of the PAN@PEG fibers was 886.1 nm. This indicates that the fibers spun by coaxial electrospinning are coarser compared with those spun by conventional electrospinning, which is consistent with the different spinning speeds of the two fibers. Higher spinning speeds result in coarser fibers. Additionally, it can also be clearly observed that the fiber membrane forms a connected three-dimensional network structure with a large porosity, which offers ample space for storing crude oil and is conducive to the rapid adsorption of viscous crude oil.

As shown in Figure 2c, the TEM image of PAN@PEG reveals that a single fiber possesses a complete core–shell structure, which prevents the leakage of phase change materials during the phase change process.

As shown in Figure 2d, the SEM image of PF@PAN@PEG reveals that polydimethylsiloxane (PDMS) is uniformly coated on the PAN@PEG fibers, while Fe_3_O_4_ nanoparticles (Fe_3_O_4_ nPs) are also dispersed and fixed onto the fiber matrix. The obtained PF@PAN@PEG exhibits a black appearance. The incorporation of Fe_3_O_4_ nPs not only improves the light absorption performance but also increases the surface roughness of the fiber membrane [39,40,41]. Furthermore, PDMS acts as an adhesive agent at the intersections of the fibers to fix their positions and further enhance the structural stability of the fiber membrane. This guarantees that PF@PAN@PEG retains a well-structured porous framework, allowing for the subsequent adsorption of viscous crude oil. As shown in Figure 2e, the EDS mapping images of Si, F, and Fe reveal that the PDMS, PFDTMS, and Fe_3_O_4_ nanoparticles are evenly distributed on the fiber surface.

**Figure 3:**
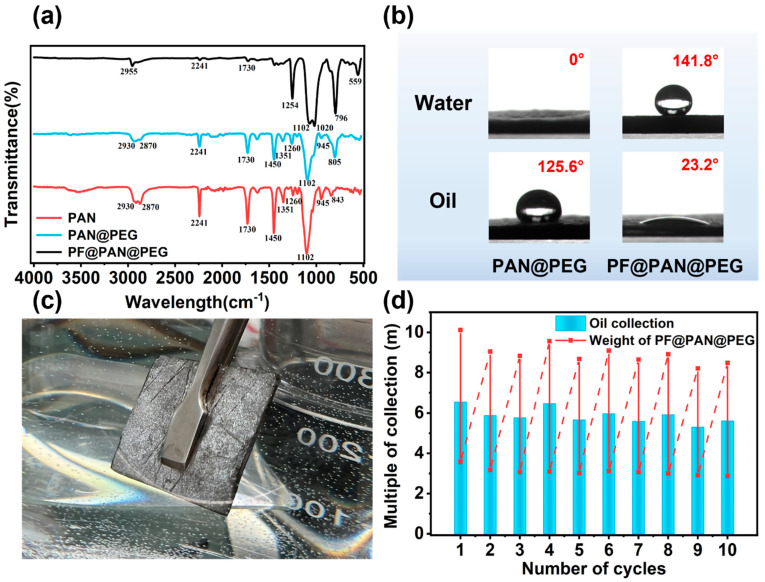
(**a**) FT-IR images of PAN, PAN@PEG, and PF@PAN@PEG; (**b**) the water and oil contact angle of PAN@PEG and PF@PAN@PEG; (**c**) photograph of PF@PAN@PEG immersed in water; (**d**) adsorption–desorption cycles of PF@PAN@PEG for crude oil collection over 10 cycles. The red line in this image represents the change of weight, where the solid line represents the deoiling process and the dotted line represents the oil absorption process.

The chemical structures of the samples were analyzed using Fourier transform infrared (FT-IR) spectroscopy. Figure 3a shows the FT-IR spectra of PAN, PAN@PEG, and PF@PAN@PEG. For PAN and PAN@PEG, the peaks at 1351 cm^−1^ (out-of-plane bending), 1450 cm^−1^ (in-plane bending), 2870 cm^−1^ (sym str), and 2930 cm^−1^ (asym str) correspond to the vibration of C-H groups. The peaks at 600–950 cm^−1^ (str) and 2241 cm^−1^ (str) are attributed to the C-C and -CN vibrations of PAN, respectively. The peak at 1729 cm^−1^ is assigned to the C=O stretching vibration, and 1102 cm^−1^ and 1260 cm^−1^ belong to the stretching vibration of C-O-C [42,43]. Since the characteristic functional groups of the added MWCNTs-OH are similar to those of PEG, the FT-IR spectra of PAN and PAN@PEG are almost identical. For PF@PAN@PEG, the peak at 559 cm^−1^ is attributed to the Fe-O vibration, the peak at 796 cm^−1^ corresponds to the Si-C stretching vibrations, and the peak at 2955 cm−1 is attributed to the C-H asymmetric stretch [44]. The peak at 1020 cm^−1^ is assigned to the asymmetric stretching vibration of Si-O-Si, and the peak at 1254 cm^−1^ corresponds to the C-F vibration [45,46]. The characteristic peaks of PDMS, PFDTMS, and Fe_3_O_4_ appear in the FT-IR spectrum of PF@PAN@PEG. Notably, the positions of the peaks have not changed, nor have any new peaks been generated, indicating that coating PAN@PEG in a hydrophobic layer involves a straightforward physical interaction rather than a chemical reaction.

### 3.2. Adsorption Performance of PF@PAN@PEG for Crude Oil

PF@PAN@PEG demonstrates both hydrophobic and oleophilic properties, which can be assessed through the material’s contact angle. The surface contact angles of PAN@PEG and PF@PAN@PEG are shown in Figure 3b, providing an intuitive representation of their wettability behaviors. The unmodified PAN@PEG does not exhibit hydrophobicity, with a water contact angle of 0°. Due to the excellent hydrophobicity of PDMS and fluorinated silane, the water contact angle of PF@PAN@PEG increases to 141.8°, while the oil contact angle is only 23.2°, which shows the selectivity of PF@PAN@PEG for oil and water. Figure 3c shows the appearance of PF@PAN@PEG submerged in water. When PF@PAN@PEG is immersed in water, a silver mirror-like air gap forms on the surface, demonstrating the excellent hydrophobicity of PF@PAN@PEG [47].

The oil storage capacity of PF@PAN@PEG was determined by crude oil adsorption tests. Firstly, the initial mass of PF@PAN@PEG was measured and denoted as m_0_. The PF@PAN@PEG was placed on the viscous crude oil for 15 min, while simultaneously being irradiated with a xenon lamp to allow for complete adsorption. Then, the PF@PAN@PEG was suspended for 10 min to allow the crude oil to drip off the sample surface until no more oil droplets were observed. The mass of the sample after this step was recorded and determined as m_1_. Next, the crude oil stored in it was extruded using a glass plate, and a 15 N force was applied to it. The mass of the sample after this was recorded and determined as m_2_. This constituted one crude oil adsorption–desorption cycle experiment. As shown in Figure 3d, the initial crude oil adsorption capacity could reach 11.65 g/g, which only decreased by 12% after 10 cycles. The results showed that PF@PAN@PEG exhibited high cycle stability in crude oil adsorption, and the adsorption performance met the typical requirements for viscous crude oil adsorption.

### 3.3. Solar Heating Conversion Properties of PF@PAN@PEG

Under environmental conditions, the high viscosity of crude oil (60,000 mPa·s) presents a significant challenge for its removal. To address this challenge, we investigated the temperature dependence of crude oil viscosity and, based on this, proposed a solar heating design to effectively recover crude oil. Additionally, phase change materials (PCMs) were incorporated to store thermal energy, thereby weakening the impact of weather fluctuations on the solar heating performance.

**Figure 4:**
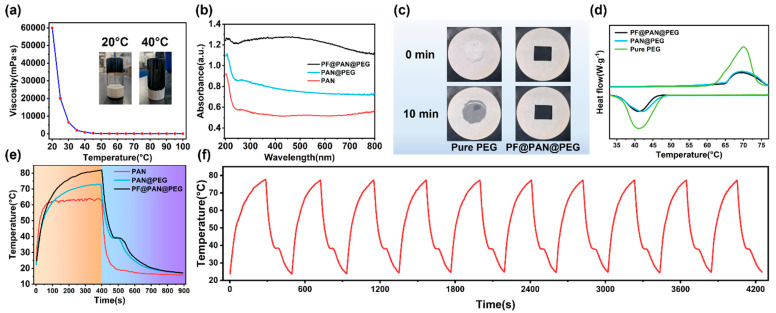
(**a**) The variation in crude oil viscosity with temperature, with images inserted showing the state of the crude oil at 20 °C and 40 °C; (**b**) UV–Vis absorption spectra of PAN, PAN@PEG, and PF@PAN@PEG; (**c**) leakage behavior of pure PEG and PF@PAN@PEG; (**d**) DSC images of PEG-10,000, PAN@PEG, and PF@PAN@PEG; (**e**) temperature change curves of PAN, PAN@PEG, and PF@PAN@PEG with light (1 kW/m^2^) and after the removal of light; (**f**) temperature change curves of PF@PAN@PEG during 10 photothermal cycles.

As shown in Figure 4a, the viscosity of crude oil decreases sharply from approximately 60,000 mPa·s at 20 °C to about 300 mPa·s at 40 °C. This indicates that increasing the temperature of crude oil can enhance its fluidity, thereby promoting its collection in the adsorbent.

The light absorption capacity of materials is a crucial factor for efficient solar energy utilization and photothermal conversion. In order to directly evaluate the light absorption performance of the samples, the UV–vis–NIR absorption spectra of PAN, PAN@PEG, and PF@PAN@PEG in the range of 200 nm to 800 nm are shown in Figure 4b. The photothermal conversion mechanisms of the composites were analyzed using ultraviolet–visible (UV–Vis–NIR) absorption spectroscopy. The light absorption capability of PAN@PEG was significantly stronger than that of PAN. This is attributed to the presence of MWCNTs-OH in both PAN and PAN@PEG. Additionally, the introduction of PEG in PAN@PEG formed an interface within the fibers. When PAN@PEG is irradiated, it undergoes additional refraction and reflection at the newly formed interface compared to PAN, which effectively increases the light absorption area of PAN@PEG and enhances its light absorption capability [48]. From the spectrum of PF@PAN@PEG, it can be observed that PF@PAN@PEG exhibits high light absorption across the 200–800 nm range, which is notably higher than that of PAN and PAN@PEG, with an average solar light absorption rate of up to 94.01%. This is primarily due to the addition of Fe_3_O_4_ nPs. Owing to the strong light absorption properties of Fe_3_O_4_, PF@PAN@PEG exhibited a wide absorption peak ranging from 250 to 700 nm, demonstrating an intense light–matter interaction, which is conducive to the absorption of light energy from the sun [49,50].

The shape stability of PF@PAN@PEG during the melting process was tested. As shown in Figure 4c, PEG undergoes a significant solid-to-liquid phase transition at high temperatures. Pure PEG-10,000 begins to melt after heating to 65 °C, and obvious liquid traces appeared in the background, indicating that PEG is unable to retain its original shape at elevated temperatures. In contrast, due to the encapsulation of PEG within the fibers during the preparation of PAN@PEG, the shape stability of PF@PAN@PEG is visibly and noticeably improved. Notably, PF@PAN@PEG exhibits excellent anti-leakage abilities, maintaining a stable shape during heating. After encapsulation, the fluidity of PEG is significantly reduced, and there are no signs of leakage after 10 min of heating.

The DSC curves for the melting and crystallization of PAN@PEG and PF@PAN@PEG are shown in Figure 4d. The specific results from the DSC curves, including the melting temperature (T_m_), crystallization temperature (T_c_), melting enthalpy (ΔH_m_), and crystallization enthalpy (ΔH_c_), are provided in Table 1. For PEG-10,000, the melting and crystallization temperatures are 70.00 °C and 41.24 °C, respectively. The latent heat values for the melting and crystallization processes, calculated from the endothermic and exothermic peaks, are 189.3 J/g and 178.2 J/g, respectively. In comparison to PAN@PEG, PF@PAN@PEG exhibits smaller peak areas and lower phase transition enthalpies due to the PDMS coating on its surface. Due to the surface effect of fibers, the larger curvature may enhance the instability of the material surface and the absorption or release of energy, thus affecting the enthalpy of the phase transition. As a result, the actual enthalpy of the samples is lower than the theoretical enthalpy (ΔH^T^, calculated using Equation (2)). The relative enthalpy efficiency (λ) represents the degree of difference between the actual enthalpy value in the energy conversion process and the theoretical enthalpy value under ideal conditions, and can be calculated by Equation (3).

(2) $$\Delta H^{T}=\Delta H_{PEG}\times r$$

(3) $$\lambda =\frac{\Delta H_{\left(sample\right)}}{\Delta H_{\left(sample\right)}^{T}}\times 100\%$$

It can also be observed from Figure 4d that the supercooling degree (T_m_ − T_c_) of PEG is relatively large, up to about 27 °C. This is because the long chain of PEG cannot be fully organized at higher temperatures during the cooling process. Therefore, it can crystallize only at a lower temperature. However, keeping the crude oil temperature around 40 °C, the crystallization temperature, is enough to enhance its fluidity, making the material effective for adsorption.

Thanks to its efficient light absorption and phase change energy storage properties, PF@PAN@PEG demonstrates exceptional photothermal conversion performance. It converts solar energy into heat through natural sunlight and stores thermal energy as latent heat during the phase change. Figure 4e presents the temperature–time curves for all samples during the photothermal conversion and release of thermal storage processes. Different samples were placed under simulated sunlight irradiation from a xenon lamp to investigate their photothermal conversion capabilities. A multi-channel data logger was used to record the temperature changes over time under a light intensity of 1.0 kW/m^2^ (1 solar unit). The ambient temperature was 12 °C, and the temperature of all samples was 25 °C without illumination. Under simulated sunlight, the temperature of PF@PAN@PEG reached 77.73 °C in 287 s, and reached 82.05 °C in 400 s, while PAN@PEG only reached 73 °C in 400 s. PAN reached 61.57 °C in only 79 s and continued to rise, reaching 63.86 °C in 400 s. This demonstrates the excellent photothermal conversion ability of the samples. It can be seen from the curves of PAN@PEG and PPF@PAN@PEG that the heating rate significantly decreases around 60 °C, indicating that the phase change material is active and that thermal energy is effectively stored in PAN@PEG and PF@PAN@PEG. Once the simulated light source was turned off, the temperature of PAN decreased rapidly, whereas the temperature decline in PAN@PEG and PF@PAN@PEG was slower, showing an obvious temperature plateau around 39.6 °C. This suggests that the thermal energy stored in PAN@PEG and PF@PAN@PEG was effectively released during the crystallization process of PEG. This confirms that the incorporation of PEG improves the sample’s ability to harness solar energy. PF@PAN@PEG is expected to sustainably reduce the viscosity and enhance the adsorption of viscous crude oil through an exothermic reaction when solar intensity decreases.

Figure 4f shows the photothermal curves of PF@PAN@PEG during 10 repeated heating–cooling cycles. Remarkably, after multiple cycles, the photothermal curves of PF@PAN@PEG showed little change, which can be attributed to the excellent phase change stability of PEG and its complete encapsulation within the fibers. This shows that PF@PAN@PEG exhibits excellent photothermal stability, making it a promising material for photothermal applications. The above results demonstrate that PF@PAN@PEG exhibits outstanding photothermal conversion, energy storage, and heat release capabilities, making it an ideal material for adsorbing high-viscosity crude oil.

**Figure 5:**
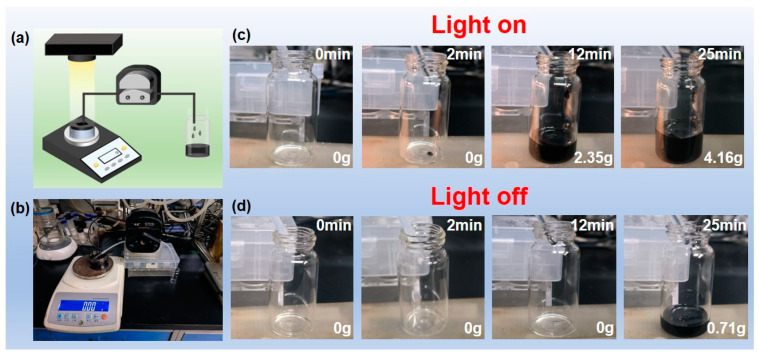
(**a**) Schematic illustration of PF@PAN@PEG for continuously collecting crude oil under simulated sunlight illumination; (**b**) device for continuous collection of crude oil on water with simulated sunlight illumination; (**c**,**d**) the crude oil absorption process with or without light.

A crucial step in practical applications is the continuous adsorption and recovery of spilled crude oil in the field during an oil leakage. Figure 5a shows the diagram of the device assembled for this purpose. Figure 5b is the self-made device assembled according to the diagram. PF@PAN@PEG was placed on the surface of the spilled oil and irradiated with a xenon lamp. A peristaltic pump was installed, and the catheter was positioned on the surface of PF@PAN@PEG, forming a “solar-heated crude oil collector” that continuously and dynamically removes crude oil from the water surface under solar irradiation.

Approximately 20 g of crude oil was added to a small bottle containing 30 mL of deionized water to simulate a crude oil spill scenario on the surface of the sea. A piece of PF@PAN@PEG was placed on the oil surface, and the simulated light source (1 solar unit) along with the peristaltic pump were activated to begin the operation of the collector. The temperature increase in PF@PAN@PEG rapidly reduced the viscosity of the surrounding viscous crude oil, enabling the heated oil to flow into the tubing through lipophilic channels in PF@PAN@PEG. Figure 5c,d shows the crude oil adsorption process with and without light exposure using this device. After 2 min of pumping, viscous crude oil collection began, and after 25 min of continuous collection, approximately 4.16 g of oil was recovered. In contrast, when the simulated light source was not turned on, crude oil began to be collected only after 12 min.

**Figure 6:**
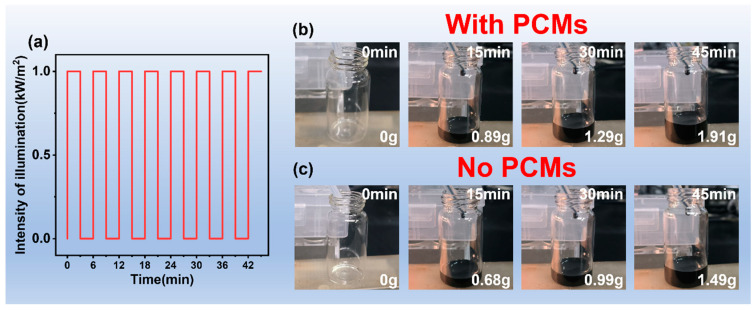
(**a**) The change in light intensity with time; (**b**) the crude oil adsorption process of adsorbents containing PCMs under the illumination changes described in (**a**); (**c**) the crude oil adsorption process of adsorbents without PCMs under the illumination changes described in (**a**).

To compare the enhancement effect of phase change materials on solar energy utilization, the modified solution outlined in Section 2.2.3 was used to modify PAN to prepare PF@PAN. Subsequently, PF@PAN and PF@PAN@PEG were, respectively, laid on the oil surface in the aforementioned simulated seawater oil spill scenario and irradiated with the light intensities shown in Figure 6a. Figure 6b,c illustrate the adsorption process of the added adsorbent materials with and without the loading of PCMs. The results show that after 45 min, PF@PAN@PEG collected 1.91 g of crude oil, while PF@PAN collected only 1.49 g. Compared to PF@PAN without the phase change material, the collection amount of PF@PAN@PEG increased by 21.99%. This further confirms that the encapsulation of PEG significantly improves the capability of the sample to utilize solar energy. PF@PAN@PEG is capable of maintaining a higher oil adsorption rate by lowering the crude oil viscosity through the release of stored latent heat when solar intensity is weakened.

## 4. Conclusions

In summary, the novel PF@PAN@PEG composite fiber exhibited outstanding performance, including excellent hydrophobicity and oleophilicity, as well as remarkable photothermal conversion and storage capabilities. Additionally, the material demonstrated high latent heat and shape stability, preventing any leakage when the phase change material was in a molten state. In this composite material, PEG served as a thermal storage material, PAN was used as a supporting carrier, and PDMS, PFDTMS, and Fe_3_O_4_ nPs were used as surface modifiers. Through coaxial electrospinning, PEG can be efficiently encapsulated into PAN fibers, and the loading rate can reach 55%. The PF@PAN@PEG composite PCM exhibits excellent encapsulation efficiency and a high latent heat value of 77.12 J/g. Among these, the Fe_3_O_4_ nPs impart exceptional light absorption abilities and an enhanced photothermal conversion performance to the composite material within the solar radiation wavelength range of 200–800 nm. Under a light intensity of 1.0 kW/m^2^, the surface temperature of the PF@PAN@PEG composite PCM can increase to 82 °C within 400 s. With its excellent photothermal conversion efficiency, good adsorption selectivity, and high porosity, the PF@PAN@PEG composite PCM demonstrated efficient recovery of viscous crude oil under solar irradiation, achieving an adsorption capacity of 11.65 g/g. In addition, the PF@PAN@PEG composite PCM exhibited excellent thermal storage capabilities. As the solar radiation intensity decreased, the material continuously maintained the high-viscous crude oil at its phase change temperature by releasing latent heat, enabling more efficient solar energy utilization and crude oil spill remediation. When the solar light intensity varied, the crude oil collection efficiency increased by 21.99% compared to when no phase change material was added. This shows the PF@PAN@PEG composite PCM’s great potential for applications in solar energy utilization and the remediation of viscous crude oil spills.

## Figures and Tables

**Table 1:** Thermal parameters of the PEG, PAN@PEG, and PF@PAN@PEG.

Sample	Melting				Crystallizing			
	T_m_ (°C)	ΔH_m_ (J g^−1^)	ΔH_m_^T^ (J g^−1^)	λ (%)	T_c_ (°C)	ΔH_c_ (J g^−1^)	ΔH_c_^T^ (J g^−1^)	λ (%)
PEG	70.00	189.3	-	-	41.24	178.2	-	-
PAN@PEG	69.50	87.66	103.93	84.34	41.98	86.21	97.84	88.11
PF@PAN@PEG	69.20	77.12	88.77	86.74	41.04	75.21	83.57	90.00

## Data Availability

The original contributions presented in this study are included in the article. Further inquiries can be directed to the corresponding authors.
